# Supplementary material for: Business sustainability of medicinal plant production under risk in the northwest region of Bangladesh: A simulation analysis
Source: PLoS One. 2025 Oct 8;20(10):e0333780. doi: 10.1371/journal.pone.0333780 (PMC12507293; doi:10.1371/journal.pone.0333780)
Supplement: S2 File — (DOCX) [file pone.0333780.s002.docx]

ECONOMIC ANALYSIS OF MEDICINAL PLANT PRODUCTION

IN SELECTED AREAS OF BANGLADESH

Department of Agribusiness and Marketing

Bangladesh Agricultural University

Mymensingh

**Questionnaire for medicinal plant producers:**

**Information for participants:** Before you begin the survey, please briefly introduce yourselves, and the study entitled “Economic analysis of medicinal plant production under different contexts in selected areas of Bangladesh”, and the rights of the participants. Please provide the participant with the Participant Information Form. [Note that the participant must be the household decision maker in medicinal plant production].

***Informed Consent:*** *Before beginning the interview, it is necessary to introduce the survey to the respondents and obtain their consent to participate. Make it clear to them that their participation in the survey is voluntary. Please read the following statement before start the survey:*

**Participation** in this survey will require up to 1 hours of your time.

**Please note:**

1. Taking part is voluntary and you can withdraw at any time without any consequences.
2. Your withdrawal will not affect you in any way. Should you wish us to destroy the records we will do so.
3. This interview and questionnaire are confidential and your privacy is respected at all times.
4. Any information that could identify you will be removed.
5. The researcher has signed a confidentiality form and cannot share information about you with any person outside the research team.
6. All information will be stored confidentially in a locked cabinet the client office for 5 years. After this time the information will be destroyed.
7. This information only use for academic research purpose with scientific publication form.

| ***Informed Verbal Consent*** | |
| --- | --- |
| *Please ask the participant (male and female) if they provide verbal consent to the participation in the study (check one box)*  [1] Yes [2] No | |
| Date of Interview: | Day: ________ Month: _______________ Year: _____________ |
| Respondent identity: |  |
| Union: |  |
| Village: |  |
| Upazila: |  |

Respondent’s types

Medicinal Plant Producer

Questionnaire number ____________________________

Name of the enumerator ____________Date: _____________Signature _____________

[*Instruction for enumerators: please interview the household head and explain the objective of the study*]

**1. Information about the respondent**

1.1 Respondent’s name…………………………………………………………………

1.2 Mobile No. (If any): ………………………

1.3 Gender (male/female/others) ………………………………

1.4 Marital status………………………………

| 1.5 | Age |  | 1.6 | Occupation |  |
| --- | --- | --- | --- | --- | --- |
| 1.7 | Education (Yrs.) |  | 1.8 | Farm experience (yrs) |  |
| 1.9 | Village name |  | 1.10 | Union |  |
| 1.11 | Upazila |  | 1.12 | District |  |

1.13 Family information

a) Total number of family members: ……. b) Male: …… c) Female: ………….

d). Working members (15-60 yrs.): …… ……(e). Male: ……. (f). Female: …….

g) School going children: ……….

1.14 In this household, who makes agricultural investment decisions? ____________ *[1=HHH, Other members (2=Men, 3= Women, 4=Men and Women together]*

**2. Land holding and tenure**

2.1 Total cultivable land by the household (dec.) : ____________

2.2 Total owned land by the household (dec.) : ____________

2.3 Land rented/shared/mortgaged out : ____________

2.4 Land rented/shared/mortgaged in : ____________

2.5 Fallow land : ____________

2.6 Homestead land : ____________

2.7 Land area under MP seedling/plant (dec) :_____________

a) owned………….decimal b) leased………...decimal c) No. of plot……….

2.8 Land use cost:

2.9 How do you inspire for MP production? ……..

**3. Gathering information related to training and financing**

3.1 Training received of MP production (give ✔mark),  Yes ***/***No.

If yes, How many times …………

1. training name……………………………………………..

training provider………………………………

1. training name……………………………………………..

training provider………………………………

3.2 Sources of finance (credit)?

Amount ………………………….… or ……….……% of total cost.

Repayment method…………………………

Interest rate……………………Installment No……………...

3.3 Do you consider your achievement knowledge on MP production is scientific and standard? Yes ***/***No.

3.4 If Yes/No, Why?

**4. Information regarding cultivation of MP**

4.1 Name of Medicinal Plant (give ✔mark):

*Aloevera*/*Ashwagandha*/*Shimulmul*

4.2 Variety ………………………………

4.3 Plot size ………………………….Decimal

4.4 Land type………………………….…………….

Available of similar land…………………………. Decimal

4.5 Soil type …………………………...…………...

Available of similar land…………………………. Decimal

4.6 Soil depth……………………inches

**5. Cropping pattern and time for survey plot**

| Pattern | crop-1 | crop-2 | crop-3 | crop-4 |
| --- | --- | --- | --- | --- |
| Present cropping pattern |  |  |  |  |
| Planting time |  |  |  |  |
| Harvesting (start)time |  |  |  |  |
| Harvesting (end)time |  |  |  |  |
| Possible cropping pattern-2 |  |  |  |  |
| Possible cropping pattern-3 |  |  |  |  |
| Possible cropping pattern-4 |  |  |  |  |
| Farmers’ opinion about better cropping pattern |  | | | |

**6. Material inputs for MP cultivation**

| Production  Practices | Time of use | | | | | | | Details | Unit | Cost (Tk.) |
| --- | --- | --- | --- | --- | --- | --- | --- | --- | --- | --- |
|  | Month  name | date or  week (give ✔mark) | | | | | Year |  |  |  |
|  |  | Date | w1 | w2 | w3 | w4 |  |  |  |  |
| *1* | *2* | *3* | *4* | *5* | *6* | *7* | *8* | *9* | *10* | *11* |
| Sowing |  |  |  |  |  |  |  | Seed Required ………….  Seed used ………Family………..purchased……… | kg | Price………tk./kg  Total cost………………. |
| Planting |  |  |  |  |  |  |  | Plant Required ……………….  total used ………Family………..purchased………  Distance of plant source (km)……  Plant age: ………..…days ;  Plant size: ………..…inches  Planting depth……………….. inches | Nos. | Price …………tk./plant  Total cost………………. |
| Fertilizer use |  | | | | | | | | | |
| Cow dung |  |  |  |  |  |  |  | …………….. times, every…………. days;  Used in each application …………….…….(kg)  Total Required………..  total used ………Family………..purchased……… | kg | Price …………tk./kg  Total cost………………. |
| Compost |  |  |  |  |  |  |  | …………….. times, every…………. days;  Used in each application …………….…….(kg)  Total Required……… total used  total used ………Family………..purchased……… | kg | Price …………tk./kg  Total cost………………. |
| Ash |  |  |  |  |  |  |  | …………….. times, every…………. days;  Used in each application …………….…….(kg)  Total Required………  total used ………Family………..purchased……… | kg | Price …………tk./kg  Total cost………………. |
| Urea |  |  |  |  |  |  |  | …………….. times, every…………. days;  Used in each application …………….…….(kg)  Total Required………  total used ……… | kg | Price …………tk./kg  Total cost………………. |
| TSP |  |  |  |  |  |  |  | …………….. times, every…………. days;  Used in each application …………….…….(kg)  Total Required………  total used ……… | kg | Price …………tk./kg  Total cost………………. |
| MP |  |  |  |  |  |  |  | …………….. times, every…………. days;  Used in each application …………….…….(kg)  Total Required………  total used ……… | kg | Price …………tk./kg  Total cost………………. |
| Gypsum |  |  |  |  |  |  |  | …………….. times, every…………. days;  Used in each application …………….…….(kg)  Total Required………  total used ……… | kg | Price ……………tk./kg  Total cost………………. |
| Lime use |  |  |  |  |  |  |  | …………….. times, every…………. days;  Used in each application …………….…….(kg)  Total Required …………….…….  total used ……… | kg | Price ……………tk./kg  Total cost………………. |
| Pesticide use |  |  |  |  |  |  |  | …………….. times, every…………. days;  Used in each application …….…….( gm/ml)  Total Required………  total used ……… | gm/ml | Price ………tk./gm/ml  Total cost………………. |
| Irrigation |  |  |  |  |  |  |  | Frequency of days…….…………. days; Method………………………  if flood irrigation, water height…………………..inches;  Source of water……………….  Total Required………  total used ……… | times | Price ………tk./time  Total cost………………. |
| Other |  |  |  |  |  |  |  | Total Required………  total used ……… |  | Price ………tk./unit  Total cost………………. |
|  |  |  |  |  |  |  |  | Total Required………  total used ……… |  | Price ………tk./unit  Total cost………………. |
|  |  |  |  |  |  |  |  | Total Required………  total used ……… |  | Price ………tk./unit  Total cost………………. |
| Harvesting: Pre-mature stage |  |  |  |  |  |  |  | Plant age……………..(days);  Plant length ………………….(inches);  if aloe-Vera, no of leaf in a plant…………………..  If harvest, how many leaf/plant need for 1 kg ………………………..(nos) |  | Price/50 kg ……….tk.  Total sale……………tk. |
| Harvesting: Peak period |  |  |  |  |  |  |  | Frequency of days …………;  harvested amount …………….kg/times  Total produced: Green………….kg; Dry…….…….kg; Dust……….kg  Market balanced………………………..  how many leaf/plant need for 1 kg …………………………..(nos) |  | Price/50 kg ……….tk.  Total sale……………tk.. |
| Harvesting: Late period |  |  |  |  |  |  |  | Frequency of days …………;  harvested amount …………….kg/times  Total produced: Green………….kg; Dry…….…….kg; Dust……….kg  Market balanced………………………..  how many leaf/plant need for 1 kg …………………………..(nos) |  | Price/50 kg ……….tk.  Total sale……………tk. |
| Harvesting closed |  |  |  |  |  |  |  | How many days required for next planting/seedling……………………days |  |  |

**7. Labor & others input requirement and Cost**

| Cost items | Labor utilization  (Mandays/hrs) | | | |
| --- | --- | --- | --- | --- |
|  | Family | Hired | Deficit (Required but not available) | Total Cost (Tk.) |
| **1. Labor requirement** |  |  |  |  |
| Collection of seed/seedling |  |  |  |  |
| Preparation of land |  |  |  |  |
| Planting, and other activities of seedling |  |  |  |  |
| Weeding, fertilizer, insecticide & water management |  |  |  |  |
| Earthing up, scratching, drain out and pruning |  |  |  |  |
| Harvesting |  |  |  |  |
| Drying |  |  |  |  |
| Crushing |  |  |  |  |
| Wage rate for one labor (Tk./day) |  |  |  |  |
| Permanent labor |  | |  |  |
| **2. Other inputs** |  |  |  |  |
| Animal draft |  |  |  |  |
| Power Tiller/Tractor |  |  |  |  |

**8. Selling information**

| Plant | Total sale (kg) | Selling  method | Selling price (Tk/kg) | Pricing method | To whom sold (incld. % of selling |
| --- | --- | --- | --- | --- | --- |
| *Aloevera* |  |  |  |  |  |
| Ashwaganda |  |  |  |  |  |
| *Shimulmul* |  |  |  |  |  |

**9. Other marketing information**

| Category | Packaging cost (Tk/unit) | | Grading cost (Tk/unit) | | Storage cost (Tk/unit) | | TC (Tk/unit) |
| --- | --- | --- | --- | --- | --- | --- | --- |
| *Aloevera* |  | |  | |  | |  |
| Ashwaganda |  | |  | |  | |  |
| *Shimulmul* |  | |  | |  | |  |
| Packaging Procedure |  |  | |  | |  | |
| Grading Procedure |  |  | |  | |  | |
| Storage methods |  |  | |  | |  | |
| Transportation Moods |  |  | |  | |  | |

**10. Household assets and income**

10.1 Household income (Yearly)

| **Income Source** | **Amount (Tk)** |
| --- | --- |
| Income from livestock & poultry |  |
| Income from fisheries/ponds |  |
| Income from wages and salaries |  |
| Income from remittance |  |
| Govt. grants or support from safety net program |  |
| Other income (specify……………………..) |  |

10.2 What is your annual total income? …………………………

10.3 What percentage of your HH income is your farm income? …………………..%

10.4 Total income coming from medicinal plants………………………

10.5 Do you have off-farm employment? (give tick)

**a.** No **b.** yes, but less than my farm income **c.** yes, more than my farm income

**10.6 Physical Asset/setting**

a) Housing type (No.): BuildingHalf-buildingTin shedStraw shed

a.1 Number of rooms in the house __________

b) Household furniture: TV Refrigerator Furniture MobileMotorbikeBy-cycle

c) Agricultural equipment: Power TillerTractorSTWThresher

d) Livestock: Cattle (No.) _____ Goats (No.) ____Poultry bird (No.) ________

e) HH has own tube-well for drinking water: Yes No

f) HH has electricity: Yes No

**11. Problems/Constraints for Harvest and Post-Harvest Activities of MP**

| **SL** | **Problem/constraint** | **Rank** |
| --- | --- | --- |
| 1 |  |  |
| 2 |  |  |
| 3 |  |  |
| 4 |  |  |
| 5 |  |  |
| 6 |  |  |
| 7 |  |  |
| 8 |  |  |
| 9 |  |  |
| *(0=very low, 1=low, 2=Moderate, 3= high, 4=very high* | | |

**12. Problems/Constraints for Marketing of MP Product**

| **SL** | **Problem/constraint** | **Rank** |
| --- | --- | --- |
| 1 |  |  |
| 2 |  |  |
| 3 |  |  |
| 4 |  |  |
| 5 |  |  |
| 6 |  |  |
| 7 |  |  |
| 8 |  |  |
| 9 |  |  |
| *(0=very low, 1=low, 2=Moderate, 3= high, 4=very high* | | |

**13. Risk related information**

13.1 Typical transplanting periods

1………………………………………………………………………… (dd/mm) 2…………………………………………………………………………

3…………………………………………….…………………………..

13.2 With the following questions we would like to learn a bit more about your risk comfort levels.

Please consider the choice you would make in the following hypothetical situation:

You will be given 150 Medicinal plants (in 5 bundles of 30 plants each) for free, to use in the coming season.

There are two types of plants, A and B, and you can choose any combination of the two that totals 5 bundles. The A and B plants have different levels of resistance to MP diseases. The A plants have potentially higher harvests but are more vulnerable to disease. If disease does not occur, the A plants will produce a harvest worth tk. 300 per bundle. However, if disease occurs (50% of the time), the A plants’ harvest is worthless (tk. 0 per bundle). The B plants are disease-resistant and always produce a harvest worth tk. 100 per bundle.

The following table illustrates the different combinations of type A and B plants that you could receive, and the value of their combined harvests based on the weather. Please check one box to indicate which combination of plants you would choose.

| I choose | Options | Bundles of 30 | | Total Taka | |
| --- | --- | --- | --- | --- | --- |
|  |  | type A plants | type B plants | If disease does not occur | If disease occur |
|  |  | Harvest: 300 tk/bundle | Harvest: 100 tk/bundle |  |  |
|  | 1 | 0 | 5 | 500 | 500 |
|  | 2 | 1 | 4 | 700 | 400 |
|  | 3 | 2 | 3 | 900 | 300 |
|  | 4 | 3 | 2 | 1100 | 200 |
|  | 5 | 4 | 1 | 1300 | 100 |
|  | 6 | 5 | 0 | 1500 | 0 |

**14. Perception & Experience with Marketing contracts**

- 1. What marketing channel(s) are you using for your MP crops (check all that apply)?
     - - 1. Direct marketing
         2. Local Wholesalers
         3. Regional Wholesalers (i.e. chain store, terminal markets, brokers etc.)
         4. Marketing Cooperatives
         5. Produce Auctions
         6. Other ______________________
  2. Have you ever participated in a marketing contract agreement for any kind of Medicinal Plant product? No Yes
  3. Have you ever participated in a marketing contract agreement for any kind of agricultural product? No Yes
  4. Would you be interested in participating in produce marketing contract agreements? No Maybe, depending on the terms Yes
  5. Please, rank the top four reasons that would **encourage** you to use a marketing contract (*1= the least important and 5 = the most important reason*)

| SL | Factors | 1 | 2 | 3 | 4 | 5 |
| --- | --- | --- | --- | --- | --- | --- |
| 1 | Reduce price risk |  |  |  |  |  |
| 2 | Opportunity to sell higher volume |  |  |  |  |  |
| 3 | Secure income |  |  |  |  |  |
| 4 | Prior experience with contracts |  |  |  |  |  |
| 5 | No need to worry about supply channels |  |  |  |  |  |
| 6 | Lower distribution cost |  |  |  |  |  |
| 7 | Access new market opportunities |  |  |  |  |  |
| 8 | Maintenance of future relationship with buyers |  |  |  |  |  |
| 9 | Bonuses for better quality |  |  |  |  |  |
| 10 | Other (Specify): |  |  |  |  |  |

- 1. Please, rank the top four reasons that would **discourage** you to use a marketing contract (*1= the least important and 5 = the most important reason*)

| SL | Factors | 1 | 2 | 3 | 4 | 5 |
| --- | --- | --- | --- | --- | --- | --- |
| 1 | Difficult to satisfy quality requirements |  |  |  |  |  |
| 2 | Unhappy with the quality terms |  |  |  |  |  |
| 3 | Unhappy with the price terms |  |  |  |  |  |
| 4 | Delivery time |  |  |  |  |  |
| 5 | Severe penalties |  |  |  |  |  |
| 6 | Method of payment |  |  |  |  |  |
| 7 | Inflexibility to pursue other markets |  |  |  |  |  |
| 8 | Not enough information about contracts |  |  |  |  |  |
| 9 | Cost of enforcement |  |  |  |  |  |
| 10 | Difficult to satisfy volume requirements |  |  |  |  |  |
| 11 | “Bad” previous experience |  |  |  |  |  |
| 12 | Other: (Specify) |  |  |  |  |  |
| 13 | Not enough land |  |  |  |  |  |

**15. Monthly price** [*try to collect from written document*]

| Medicinal Plant | Monthly price (Tk./kg) | | | | | | | | | | | |
| --- | --- | --- | --- | --- | --- | --- | --- | --- | --- | --- | --- | --- |
|  | Jan | Feb | Mar | Apr | May | Jun | Jul | Aug | Sep | Oct | Nov | Dec |
|  | 1 | 2 | 3 | 4 | 5 | 6 | 7 | 8 | 9 | 10 | 11 | 12 |
| *Aloevera* |  |  |  |  |  |  |  |  |  |  |  |  |
| Ashwaganda |  |  |  |  |  |  |  |  |  |  |  |  |
| *Shimulmul* |  |  |  |  |  |  |  |  |  |  |  |  |
