# Supplementary material for: Business sustainability of medicinal plant production under risk in the northwest region of Bangladesh: A simulation analysis
Source: PLoS One. 2025 Oct 8;20(10):e0333780. doi: 10.1371/journal.pone.0333780 (PMC12507293; doi:10.1371/journal.pone.0333780)
Supplement: S3 File — (DOCX) [file pone.0333780.s003.docx]

**Department of Agribusiness and Marketing**

Bangladesh Agricultural University, Mymensingh

**Economic analysis of medicinal plant production under different contexts in selected areas of Bangladesh**

**Questionnaire for medicinal plant producers:**

**Information for participants:** Before you begin the survey, please briefly introduce yourselves, and the study entitled “Economic analysis of medicinal plant production under different contexts in selected areas of Bangladesh”, and the rights of the participants. Please provide the participant with the Participant Information Form. [Note that the participant must be the household decision maker in medicinal plant production].

***Informed Consent:*** *Before beginning the interview, it is necessary to introduce the survey to the respondents and obtain their consent to participate. Make it clear to them that their participation in the survey is voluntary. Please read the following statement before start the survey:*

**Participation** in this survey will require up to 1 hours of your time.

**Please note:**

1. Taking part is voluntary and you can withdraw at any time without any consequences.
2. Your withdrawal will not affect you in any way. Should you wish us to destroy the records we will do so.
3. This interview and questionnaire are confidential and your privacy is respected at all times.
4. Any information that could identify you will be removed.
5. The researcher has signed a confidentiality form and cannot share information about you with any person outside the research team.
6. All information will be stored confidentially in a locked cabinet the client office for 5 years. After this time the information will be destroyed.
7. This information only use for academic research purpose with scientific publication form.

| ***Informed Verbal Consent*** | |
| --- | --- |
| *Please ask the participant (male and female) if they provide verbal consent to the participation in the study (check one box)*  [1] Yes [2] No | |
| Date of Interview: | Day: ________ Month: _______________ Year: _____________ |
| Respondent identity: |  |
| Union: |  |
| Village: |  |
| Upazila: |  |
