# Supplementary material for: Business sustainability of medicinal plant production under risk in the northwest region of Bangladesh: A simulation analysis
Source: PLoS One. 2025 Oct 8;20(10):e0333780. doi: 10.1371/journal.pone.0333780 (PMC12507293; doi:10.1371/journal.pone.0333780)
Supplement: Attachment — Submitted filename: Response to Reviewers_Final.docx [file pone.0333780.s005.docx]

**PONE-D-25-10568**

**Title:**	Business sustainability of medicinal plants production under risk in the northwest region of Bangladesh: a simulation analysis

**Response to reviewers' comments:**

Thank you for acknowledging the scientific quality of our manuscript. We also appreciate the reviewer’s focus on the technical soundness and the relationship between the data and conclusions. In response to reviewers' comments, we have revised the respective sections. We followed the comments raised by the reviewers and also followed the journal guidelines.

| **Journal requirements** | **Response/Possible adjustment** |
| --- | --- |
| Please ensure that your manuscript meets PLOS ONE's style requirements, including those for file naming. | We followed the journal requirements that describe in the web link. |
| You indicated that ethical approval was not necessary for your study. We understand that the framework for ethical oversight requirements for studies of this type may differ depending on the setting and we would appreciate some further clarification regarding your research. Could you please provide further details on why your study is exempt from the need for approval and confirmation from your institutional review board or research ethics committee (e.g., in the form of a letter or email correspondence) that ethics review was not necessary for this study? Please include a copy of the correspondence as an ""Other"" file. | Because all human involvement was guaranteed in accordance with local laws and institutional concerns, this study did not require ethical approval. Besides, this study was carried out in accordance with the Helsinki Declaration's guidelines for human subjects research. Additionally, since there was no institutional ethical board to authorize the social science study, there was no opportunity to apply for Research Ethics Board (REB) approval. Due to this academic limitation and local legislation, this study is exempt from the need for approval and confirmation from Institutional research ethics committee.  The study relied on publicly available data, secondary sources, and information voluntarily provided by farmers through informal interviews and field visits. As such, the scope of the research did not require prior ethical clearance under existing national research guidelines for non-clinical, economic studies. Nevertheless, all activities were conducted with respect for local norms, with verbal consent obtained from participants, and in alignment with the ethical standards for socio-economic research. We are committed to upholding high ethical standards in all aspects of our research. |
| Please note that PLOS ONE has specific guidelines on code sharing for submissions in which author-generated code underpins the findings in the manuscript. In these cases, we expect all author-generated code to be made available without restrictions upon publication of the work. Please review our guidelines at https://journals.plos.org/plosone/s/materials-and-software-sharing#loc-sharing-code and ensure that your code is shared in a way that follows best practice and facilitates reproducibility and reuse. | Data are made available after requesting to corresponding author. There is no code available for sharing. . |
| Please include captions for your Supporting Information files at the end of your manuscript, and update any in-text citations to match accordingly. Please see our Supporting Information guidelines for more information: http://journals.plos.org/plosone/s/supporting-information. | The top page of survey questionnaire is attached as supporting information 1 in the manuscript. |
| **Reviewer comments** |  |
| Is the manuscript technically sound, and do the data support the conclusions?  The manuscript must describe a technically sound piece of scientific research with data that supports the conclusions. Experiments must have been conducted rigorously, with appropriate controls, replication, and sample sizes. The conclusions must be drawn appropriately based on the data presented.  Reviewer #1: Partly  Reviewer #2: Yes | We thank both reviewers for their assessments of our manuscript and appreciate their thoughtful feedback.  **Reviewer #1:** We appreciate the reviewer’s focus on the technical soundness and the relationship between the data and conclusions. We would like to confirm that the methodologies used in the study are standard and widely accepted in the field. We have clearly described the experimental/analytical procedures in the Methods section, ensuring reproducibility. The conclusions are directly drawn from the results, without over-interpretation. For further clarify this, we have revised the Discussion section to more explicitly link specific results to the conclusions.  **Reviewer #2:** We are grateful for this positive evaluation. |
| Has the statistical analysis been performed appropriately and rigorously?  Reviewer #1: Yes  Reviewer #2: Yes | We thank both reviewers for recognizing that the statistical analyses used in our study was performed appropriately and rigorously. |
| Have the authors made all data underlying the findings in their manuscript fully available?  The [PLOS Data policy](http://track.editorialmanager.com/CL0/http:%2F%2Fwww.plosone.org%2Fstatic%2Fpolicies.action%23sharing/1/010f0196ce4813f6-d7bb005a-514e-4fe5-bf64-5558921fce0a-000000/704lopkDbDc-tsJ1wy-1VjHJ7qkq--aXvVUNZKdkBjE=211) requires authors to make all data underlying the findings described in their manuscript fully available without restriction, with rare exception (please refer to the Data Availability Statement in the manuscript PDF file). The data should be provided as part of the manuscript or its supporting information, or deposited to a public repository. For example, in addition to summary statistics, the data points behind means, medians and variance measures should be available. If there are restrictions on publicly sharing data—e.g. participant privacy or use of data from a third party—those must be specified.  Reviewer #1: Yes  Reviewer #2: Yes | We thank both reviewers for confirming that the data underlying our findings have been made fully available in accordance with PLOS ONE’s data sharing policy. |
| Is the manuscript presented in an intelligible fashion and written in standard English?  PLOS ONE does not copyedit accepted manuscripts, so the language in submitted articles must be clear, correct, and unambiguous. Any typographical or grammatical errors should be corrected at revision, so please note any specific errors here.  Reviewer #1: Yes  Reviewer #2: Yes | We thank both reviewers for their positive assessment of the clarity and quality of the language used in our manuscript. We have carefully reviewed the manuscript again to ensure it remains free of typographical or grammatical errors. |
| **Reviewer #1** |  |
| The manuscript id: PONE-D-25-10568 entitled: "Business Sustainability of Medicinal Plants Production under Risk in the Northwest Region of Bangladesh: A Simulation Analysis" has been reviewed. In my opinion there is no problem with its scientific content but I think this manuscript subject is not qualified to be published in the PLOS ONE but you are encouraged to submit this paper to another Journal which better covers the subject. Best regards | We appreciate the reviewer’s acknowledgement that there are no issues with the scientific content of the manuscript. With due respect, we would like to clarify how our study aligns with the scope and objectives of PLOS ONE.  Our manuscript presents:   - A data-driven analysis combining economic simulation with sustainability risk assessment. - A real-world case study from an under-researched region (Northwest Bangladesh) that informs both sustainable agricultural practices and rural economic development. - A modeling approach that is reproducible and applicable in other geographic contexts or sectors facing similar risk-based sustainability challenges.   We believe these contributions align within the interdisciplinary scope of PLOS ONE. We also have revised the manuscript to better more highlight its relevance to the journal's audience.  We hope based on these ground and Journal scope (**primary research** that contributes to the base of scientific knowledge, including interdisciplinary, replication studies, and negative or null results) this manuscript is best fitted in the PLOS ONE. |
| Reviewer #2: |  |
| This is an interesting study, which reports the simulation analysis regarding the sustainability of medicinal plant business in the northwest Bangladesh. Overall, the study is well-organized and well-written. However, following are some aspects, which need to be addressed for improved presentation, scientific rigor and validity of the manuscript. | Thank you for acknowledging the scientific quality of our manuscript. We have incorporated the comments as per suggestions. |
| Abstract: Please revise the last sentence. | The last sentence has been revised based on data presented. |
| Introduction: It’s a bit lengthy. Shorten the introduction. Introduction should not be starting locally from Bangladesh. Remove the first paragraph and use some of its part to be presented as just 1-2 sentences at the end of second paragraph. Its better to not start sentence with an abbreviation (throughout the manuscript). | Thank you for your comments. We accepted this suggestion and shortened the introduction. |
| L 97. Please remove grammatical errors from the manuscript. | Grammatical errors have been removed. |
| L 100-107. Revise to present a single sentence by stating the objective of this study instead of numbering the research questions. | Revised as of comments. |
| Remove L 108-110. | Removed as of comments. |
| Methodology: L 114. Sustainability “of” instead of “in” | Revised as of comments. |
| Fig 2. As the study areas are restricted to two yellow points, is this a true representation of the complete northwest region? | The northwest region of Bangladesh includes several other districts besides Natore and Bogura, such as Rajshahi, Naogaon, Pabna, Rangpur, Dinajpur, and more. Although the study is confined to Natore and Bogura districts, these areas are widely recognized as key hubs for medicinal plant cultivation in the northwest region of Bangladesh. Both districts share similar agro-ecological, cultural, and socio-economic characteristics with neighboring districts in the region. As such, they can be considered a reasonably representative sample of the broader northwest context, particularly in terms of medicinal plant cultivation practices. |
| Please use past tense while describing the methodology of the currents study. | Methodology of the currents study has been revised as of comments. |
| L 168. Please revise this sub-heading | Revised as of comments. |
| L 205. Why no approval of any regulatory/local law authority or research organization/institution was obtained to conduct this study?  Although no human subjects were directly involved, the study focused on medicinal plants business and publication of these findings will be directly associated with the business related to human and animal health. Therefore, at least permission to conduct this study should be mentioned with relevant approval number from the relevant institution/ regulatory authority for the validity of the study. | We sincerely thank the reviewer for this thoughtful observation. While no formal regulatory or institutional approval was obtained for this study, we would like to clarify that the research focused exclusively on the economic and financial aspects of medicinal plant (MP) production at the farm level and did not involve any experimental interventions, clinical trials, or the use of human or animal subjects.  The study relied on publicly available data, secondary sources, and information voluntarily provided by farmers through informal interviews and field visits. As such, the scope of the research did not require prior ethical clearance under existing national research guidelines for non-clinical, economic studies. Nevertheless, all activities were conducted with respect for local norms, with verbal consent obtained from participants, and in alignment with the ethical standards for socio-economic research.  We acknowledge the importance of transparency in research related to human and animal health, and we have now included a statement in the manuscript clarifying that no formal approval was required given the nature and scope of the study. We are committed to upholding high ethical standards in all aspects of our research. Besides, as described in the manuscript, in developing countries like Bangladesh, most of the institutions have no ethical clearance board. In Bangladesh Agricultural University and in the Faculty of Agricultural Economics and Rural Sociology has recently formed (mid-June/2024) an ethical board for taking approval before data collection. The data of this research were collected earlier 2018-2020. Therefore, there is no scope to apply for ethical clearance.  In addition, we also described in the manuscript, we were taken verbal permission from the participants and its’ data will be used only research purpose and publication.  Based on this ground, we exempt ethical approval. |
| Table 1. How it was decided to use these assumptions, As these values may affect the outcomes, it is important to mention that how these assumption values including interest and discount rates were decided. | Table-1 represents the following key assumptions, which were used in the Net Present Value (NPV) analysis to model the investment viability and financial performance of MP production at the farm level. These assumptions allowed for a standardized and realistic evaluation of long-term returns and risks associated with MP production using NPV methodology:  **Farm size:** The NPV analysis was based on a standard 1-hectare farm, ensuring consistency across different farm types and simplifying comparison of economic outcomes.  **Ownership:** It was assumed that the farmer owned 100% of the land, implying no rental or lease costs were considered in the model.  **Loan length:** The analysis assumed a one-year loan term, reflecting short-term borrowing often used for operational capital in agriculture.  **Flat rate of interest:** A fixed annual interest rate of 9% was applied to borrowed capital, representing prevailing rural credit rates during the study period. This is also Bank interest rate prevailing during the data collection period.  **Initial equity:** It was assumed that farmers had no initial equity, meaning the entire investment was financed through credit, to simulate a conservative scenario.  **Life cycle:** A 10-year investment horizon was considered for the analysis, matching the productive life cycle of perennial MP species.  **Discount rate:** An 8% discount rate was used to reflect the time value of money and the opportunity cost of capital in agricultural investments. The discounting factor is related to the interest rate and hence it is considered 8%. |
| L 267. On which basis authors calculated these values. Please indicate the relevant sources of values used for calculation. | Sources of Stochastic Risk Variables Used in the Simulation Model:  **Yield:** Field survey data (2018-2020); validated against regional agricultural statistics and previous MP yield studies (e.g., [28], [60], [61]).  **Producer's price:** Local market surveys, trader interviews, and farm gate price reports collected during field visits (2018-2020); supported by Bangladesh Bureau of Statistics (BBS) and Ministry of Agriculture (MoA) reports [60], [61], [70].  **Seed price:** Data from local farmers, input suppliers, and Agricultural Extension Offices (UAO) reports; triangulated with previous studies on input costs [28], [34], [60].  **Market absorption:** Estimated from farmer responses on post-harvest sales volumes and price realization; confirmed via interviews with local buyers and cooperatives [28], [34], [60]. |
| The results of present study should be presented in past tense. Please check complete manuscript for grammatical mistakes according to each section. | Revised as of comments. |
| Another but major concern regarding results and discussion section is the lack of comparison of the current results with previous studies conducted in Bangladesh, neighboring countries, or anywhere around the world, which should be incorporated with relevant explanations of any differences in findings. | As mentioned in the introduction section, so per our google search history, this type of research is rare in the literature. Only two studies ([14], [29]) were found and we made comparison of this study finding to the earlier two studies. |
| Tables. Please add the basis of the calculations with relevant references in methodology and remove the footnote of authors own calculation from result tables. | Revised as of comments. |
| Revise the first sentence of conclusion. | Revision has been done in the first sentence of conclusion. |
| Avoid using abbreviations in headings, table and figure captions. | Revised as of comments. |
