# Supplementary material for: Business sustainability of medicinal plant production under risk in the northwest region of Bangladesh: A simulation analysis
Source: PLoS One. 2025 Oct 8;20(10):e0333780. doi: 10.1371/journal.pone.0333780 (PMC12507293; doi:10.1371/journal.pone.0333780)
Supplement: Attachment — Submitted filename: Response_to_Reviewers_Final_auresp_2.docx [file pone.0333780.s006.docx]

**PONE-D-25-10568**

**Title:**	Business sustainability of medicinal plants production under risk in the northwest region of Bangladesh: a simulation analysis

**Response to reviewers' comments:**

Thank you for acknowledging the scientific quality of our manuscript. We also appreciate the reviewer’s focus on the technical soundness and the relationship between the data and conclusions. In response to reviewers' comments, we have revised the respective sections. We followed the comments raised by the reviewers and also followed the journal guidelines.

| **Journal requirements** | **Response/Possible adjustment** |
| --- | --- |
| If the reviewer comments include a recommendation to cite specific previously published works, please review and evaluate these publications to determine whether they are relevant and should be cited. There is no requirement to cite these works unless the editor has indicated otherwise. | Thank you for your suggestions. |
| **Reviewer's Responses to Questions** |  |
| **Comments to the Author** |  |
| 2. Is the manuscript technically sound, and do the data support the conclusions?  The manuscript must describe a technically sound piece of scientific research with data that supports the conclusions. Experiments must have been conducted rigorously, with appropriate controls, replication, and sample sizes. The conclusions must be drawn appropriately based on the data presented.  Reviewer #3: Partly  Reviewer #4: Yes | Thanks the both reviewers for their thoughtful comments and suggestions. |
| 3. Has the statistical analysis been performed appropriately and rigorously?  Reviewer #3: Yes  Reviewer #4: Yes | Thanks |
| 4. Have the authors made all data underlying the findings in their manuscript fully available?  The [PLOS Data policy](http://track.editorialmanager.com/CL0/http:%2F%2Fwww.plosone.org%2Fstatic%2Fpolicies.action%23sharing/1/010f0198077c3714-dc937803-aab1-4270-8329-5f4c0130b518-000000/Pc4MsuwxgoMB7Rip-WnzExn3qvKLjrM2uRPaXoErLAM=219) requires authors to make all data underlying the findings described in their manuscript fully available without restriction, with rare exception (please refer to the Data Availability Statement in the manuscript PDF file). The data should be provided as part of the manuscript or its supporting information, or deposited to a public repository. For example, in addition to summary statistics, the data points behind means, medians and variance measures should be available. If there are restrictions on publicly sharing data—e.g. participant privacy or use of data from a third party—those must be specified.  Reviewer #3: No  Reviewer #4: Yes | We uploaded the data file in the submission process. |
| 5. Is the manuscript presented in an intelligible fashion and written in standard English?  PLOS ONE does not copyedit accepted manuscripts, so the language in submitted articles must be clear, correct, and unambiguous. Any typographical or grammatical errors should be corrected at revision, so please note any specific errors here.  Reviewer #3: Yes  Reviewer #4: Yes | Thanks |
| 6. Review Comments to the Author |  |
| Reviewer #3: This is an interesting manuscript, a good research question, sound methodology, and comprehensive research design. The authors have improved the manuscript based of first reviewers' reports (I was not a part of the first review round).  My main concern with the manuscript is that it is very unclear how the 196 interviews, which were mentioned as the primary source of data in the abstract, relate to the presented findings. There is limited or no information on the interview design (which questions were asked) nor on the interview results (which answers were given). Most of the modeling input data is attributed to secondary sources rather than the interviews themselves. I would expect a wide range of interview responses based on these 196 interviews with individual farms. This would require a dedicated Results section where these descriptive results are presented, and consequently, how they are then used (aggregated?) in the general modeling exercise. If modeling was mainly done based on other sources, the manuscript methodology and abstract should be written to reflect so. If 196 interviews were performed using the same interview design and protocol, these results would be of much interest to the academic community and should thus be given more attention. For example, were your three main risk indicators qualified based on the farmer interviews or were they selected a priori? Etc.  A minor suggestion would be to move all the discussion of results that are currently integrated in the Results section to a dedicated Discussion section, rather than a Conclusion. | Thank you for your appreciation.  Thanks for your concern about the interview and the questions. This manuscript is based on the survey questionnaire. We used pre-tested questionnaire for collecting necessary data from the respondents. The questionnaire is attached in the supporting file. You may look what types of questions are asked during the survey. The questionnaire is transferred into the excel sheet. The data file is also found in the submission system. In the modelling purpose we take few assumptions that also explained in the previous version. For your more understanding, we also present them here:  These assumptions allowed for a standardized and realistic evaluation of long-term returns and risks associated with MP production using NPV methodology:  **Farm size:** The NPV analysis was based on a standard 1-hectare farm, ensuring consistency across different farm types and simplifying comparison of economic outcomes.  **Ownership:** It was assumed that the farmer owned 100% of the land, implying no rental or lease costs were considered in the model.  **Loan length:** The analysis assumed a one-year loan term, reflecting short-term borrowing often used for operational capital in agriculture.  **Flat rate of interest:** A fixed annual interest rate of 9% was applied to borrowed capital, representing prevailing rural credit rates during the study period. This is also Bank interest rate prevailing during the data collection period.  **Initial equity:** It was assumed that farmers had no initial equity, meaning the entire investment was financed through credit, to simulate a conservative scenario.  **Life cycle:** A 10-year investment horizon was considered for the analysis, matching the productive life cycle of perennial MP species.  **Discount rate:** An 8% discount rate was used to reflect the time value of money and the opportunity cost of capital in agricultural investments. The discounting factor is related to the interest rate and hence it is considered 8%.  Sources of Stochastic Risk Variables Used in the Simulation Model:  **Yield:** Field survey data (2018-2020); validated against regional agricultural statistics and previous MP yield studies (e.g., [28], [60], [61]).  **Producer's price:** Local market surveys, trader interviews, and farm gate price reports collected during field visits (2018-2020); supported by Bangladesh Bureau of Statistics (BBS) and Ministry of Agriculture (MoA) reports [60], [61], [70].  **Seed price:** Data from local farmers, input suppliers, and Agricultural Extension Offices (UAO) reports; triangulated with previous studies on input costs [28], [34], [60].  **Market absorption:** Estimated from farmer responses on post-harvest sales volumes and price realization; confirmed via interviews with local buyers and cooperatives [28], [34], [60].  The three indicators used here is based on priori experience and reality that farmer faced in their farming.  Thanks for the suggestions. We moved all discussion in the discussion section. |
| Reviewer #4: This paper presents a technically rigorous study that explores an important issue in agricultural sustainability. The authors used a scientific methodology, combining Monte Carlo simulations and ROV, to assess the commercial sustainability of medicinal plant production under uncertainty. The data source is reliable, coming from a valid survey of 197 farmers, and supports the conclusions presented in the paper. The statistical analysis is rigorous, and the advanced methods used add credibility to the findings.  Although the paper is well presented and written in standard English, some minor areas could be improved. The introduction section could be reorganized and optimized to present the research question more clearly. In addition, the literature review should be further expanded and presented as a separate section after the introduction section, with an additional discussion of the research gaps in existing research and an emphasis on the theoretical contribution of the study.  Finally, the discussion section is weak and could be improved by a deeper extension of the analysis of the findings, their significance for agricultural development in countries including Bangladesh, possible policy implications, and future research directions. Strengthening these sections would enhance the theoretical and practical contributions of the paper.  Overall, the paper is technically rigorous, the statistical analysis is appropriate, and the data support the conclusions. The paper is clear, but further development in some areas would significantly improve its clarity and impact. | Thank you for your positive feedback.  The introduction is slightly modified following your comments and suggestions.  We incorporated literature review in the introduction section which is well practiced in scientific arena. At this stage, we think the introduction section is well organized and clearly present the research gaps. Therefore, we keep them as together.  We improved the discussion section with following your suggestions.  Thanks for your feedback and time spent for reading this manuscript. |
