# Supplementary material for: Business sustainability of medicinal plant production under risk in the northwest region of Bangladesh: A simulation analysis
Source: PLoS One. 2025 Oct 8;20(10):e0333780. doi: 10.1371/journal.pone.0333780 (PMC12507293; doi:10.1371/journal.pone.0333780)
Supplement: Attachment — Submitted filename: Response to Reviewers.docx [file pone.0333780.s007.docx]

**PONE-D-25-10568**

**Title:**	Business sustainability of medicinal plants production under risk in the northwest region of Bangladesh: a simulation analysis

**Response to reviewers' comments:**

Thank you for acknowledging the scientific quality of our manuscript. We also appreciate the reviewer’s focus on the technical soundness and the relationship between the data and conclusions. In response to reviewers' comments, we have revised the respective sections. We followed the comments raised by the reviewers and also followed the journal guidelines.

| **Journal requirements** | **Response/Possible adjustment** |
| --- | --- |
| 1. If the reviewer comments include a recommendation to cite specific previously published works, please review and evaluate these publications to determine whether they are relevant and should be cited. There is no requirement to cite these works unless the editor has indicated otherwise. | Thank you for your suggestions. |
| 2. Please review your reference list to ensure that it is complete and correct. If you have cited papers that have been retracted, please include the rationale for doing so in the manuscript text, or remove these references and replace them with relevant current references. Any changes to the reference list should be mentioned in the rebuttal letter that accompanies your revised manuscript. If you need to cite a retracted article, indicate the article’s retracted status in the References list and also include a citation and full reference for the retraction notice. |  |
| **Reviewer's Responses to Questions** |  |
| **Comments to the Author** |  |
| 1. 1. If the authors have adequately addressed your comments raised in a previous round of review and you feel that this manuscript is now acceptable for publication, you may indicate that here to bypass the “Comments to the Author” section, enter your conflict of interest statement in the “Confidential to Editor” section, and submit your "Accept" recommendation.  Reviewer #3: (No Response)  Reviewer #4: All comments have been addressed | Thanks |
| 2. Is the manuscript technically sound, and do the data support the conclusions?  The manuscript must describe a technically sound piece of scientific research with data that supports the conclusions. Experiments must have been conducted rigorously, with appropriate controls, replication, and sample sizes. The conclusions must be drawn appropriately based on the data presented.  Reviewer #3: Partly  Reviewer #4: Yes | Thanks the both reviewers for their thoughtful comments and suggestions. |
| 3. Has the statistical analysis been performed appropriately and rigorously?  Reviewer #3: Yes  Reviewer #4: Yes | Thanks |
| 4. Have the authors made all data underlying the findings in their manuscript fully available?  The [PLOS Data policy](http://track.editorialmanager.com/CL0/http:%2F%2Fwww.plosone.org%2Fstatic%2Fpolicies.action%23sharing/1/010f01985ad1f591-a4b2ba4a-dfc8-4cd1-a4c3-0b843a568c11-000000/NZqMldYayU8p2KFeytUWGBvbr09cgGyNrOBEXH6d6Jo=221) requires authors to make all data underlying the findings described in their manuscript fully available without restriction, with rare exception (please refer to the Data Availability Statement in the manuscript PDF file). The data should be provided as part of the manuscript or its supporting information, or deposited to a public repository. For example, in addition to summary statistics, the data points behind means, medians and variance measures should be available. If there are restrictions on publicly sharing data—e.g. participant privacy or use of data from a third party—those must be specified.  Reviewer #3: Yes  Reviewer #4: Yes |  |
| 5. Is the manuscript presented in an intelligible fashion and written in standard English?  PLOS ONE does not copyedit accepted manuscripts, so the language in submitted articles must be clear, correct, and unambiguous. Any typographical or grammatical errors should be corrected at revision, so please note any specific errors here.  Reviewer #3: Yes  Reviewer #4: Yes | Thanks |
| 6. Review Comments to the Author |  |
| 6. Review Comments to the Author  Please use the space provided to explain your answers to the questions above. You may also include additional comments for the author, including concerns about dual publication, research ethics, or publication ethics. (Please upload your review as an attachment if it exceeds 20,000 characters)  Reviewer #3: I appreciate the authors response to my first review questions. And while I appreciate the substantial supporting information, including the survey documents and collected data, the manuscript itself should be able to stand on its own, containing the most important findings and results of the study without having to go into the SI. I still find an inconsistency in the structure and presentation of the manuscript. The abstract mentions the study method was data collection through 196 surveys which were then used in NPV and further analysis. However, the results of the surveys seem to be presented numerically in the Methods (Table 2, average scores on yield, prices, etc.). While these factors are inputs to the analysis, they are not part of the Methods but they are part of the study's results. They were not known before the study started. I would suggest moving these numbers to a first section in the Results, where I would also expect (as by my previous comments) to have a more substantial overview of what was found in the surveys, rather than only summary statistics. That is, you have a 15-page questionnaire instrument, used across a wide range of farmers in different geographical areas. A descriptive section based on the most relevant background information and outcome data of interest would help readers better understand and transition into the findings of the NPV. | Thank you for your appreciation.    Thanks for your further comments on the method. We modified the Table 2 in the method section. We also add brief description of questionnaire.  We also presented an overview of survey findings in the result section. |
| Reviewer #4: The author has addressed my concerns. I think this paper has been well-written and can be accepted for this journal. | Thank you for your positive feedback. |
